# Supplementary material for: Satisfaction of chronic patients with community-based health insurance schemes and related factors: Explanatory sequential mixed methods study
Source: PLOS Glob Public Health. 2024 Aug 7;4(8):e0003374. doi: 10.1371/journal.pgph.0003374 (PMC11305578; doi:10.1371/journal.pgph.0003374)
Supplement: Attachment — Submitted filename: The community based mixed methods comments.docx [file pgph.0003374.s001.docx]

**Satisfaction with Community-Based Health Insurance Scheme and Its Associated Factors among Chronic Disease Follow-up Patients in East Gojjam Zone Hospitals, Northwestern Ethiopia, 2022: Mixed Method Study**

**Manuscript Number: PGPH-D-23-01081**

This is a mixed methods study with both quantitative (cross sectional ) and qualitative (qualitative description ) analysis

This is an important study that seeks to determine the satisfaction of patients with chronic diseases receiving heath care through health schemes since this my determine the quality of life of these patients

**A Quality of the mixed methods study**

1 This study through mixed methods will contribute e new knowledge that will improve on the universal health coverage services and new policy to that effect in that region of Ethiopia

2 The problem of poor access to health services is noteworthy

3 A theoretical framework should be included in the write up.

4The research question compiles with the mixed methods

5 Both the qualitative and quantitative methods were performed

6 Both sampling for quantitative and qualitative methods was done.

7 I did not find a plan of the integration of the two analyses

8 The discussion is in keeping with the results and in coherence with previous finding

**B General Comments**

1. The title is too long I suggest a reduction in zone location

2. The English style is poor therefore reduces coherence leading to difficulty for the reader to understand. I suggest that the authors seek for assistance from an English native

3. In the introduction the authors need to include something on the scheme coverage and how it relates to the social demographic factors since in the methods am struggling to understand the independent variables**, Enroller’s knowledge of health insurance benefit package relates to the background and objective**.

4. Did the authors use focus group discussions for data collection? It was not clearly brought out in the methods

5. The age group of the study seems variable in the text. Table 1 shows the age categories. What was the mean age of the study since age became a limitation

6. The full sample size calculation may not be necessary in the methods

7. The ethical approval subtitle font is different from the rest

8. After the conclusion the authors should write a clear statement on what they recommend

9. No evidence of Supplementary data. Authors should avail data
