# Supplementary material for: Satisfaction of chronic patients with community-based health insurance schemes and related factors: Explanatory sequential mixed methods study
Source: PLOS Glob Public Health. 2024 Aug 7;4(8):e0003374. doi: 10.1371/journal.pgph.0003374 (PMC11305578; doi:10.1371/journal.pgph.0003374)
Supplement: Attachment — Submitted filename: Responses to Editors and Reviewers.docx [file pgph.0003374.s002.docx]

**Dear Editors and Reviewers in PLOS Global Public Health**

I would like to thank you for providing constructive comments and for devoting time to review the paper with manuscript number, **‘PGPH-D-23-0108’**, entitled as ‘**Satisfaction of Chronic Patients with Community-Based Health Insurance Scheme and Related Factors: Explanatory Sequential Mixed Methods Study**’**.** Most comments have been incorporated in the paper and some responses to editors and reviewers have been presented below.

1. In terms of sample size calculations, we had trouble finding a study with chronic patients as the study population in Ethiopia and Africa, at large. Therefore, we have only taken chronic patients who are household heads. To determine our sample size, we employed a local study that included household heads, whether or not they were chronic patients. The comparison was also difficult. It was also cited as a major limitation.
2. An explanatory sequential mixed methods study was employed, which involved collecting and analyzing quantitative data first, then qualitative data. The level of satisfaction can be measured. CBHI satisfaction is of a qualitative nature. The outcomes of this quantitative study are validated by adding qualitative data as a method of triangulation. Additionally, it provides in-depth understanding of the problem that was found.
3. This study was limited in that it was unable to take into account a number of factors, including the attitude of the health care providers toward insurance scheme membership, the trust that patients have in the contracted health facilities, the standard of healthcare services, and the accessibility and availability of health care. But by themselves, these variables can be topics of study that encompass a wider field of study.
4. Each hospital, waiting times, queue management techniques, and complaint handling procedures have been assessed objectively. The managing crew of the card room recorded the starting time on the day of data collection at arrival to measure the waiting times. There were officers assigned in each system of complaint handling and queue managing process. The measured the absence or presence of complaint handling officers, or whether the client report the complaint. The same is true for queue managing officers.
5. Because most chronic patients were unable to recall and describe the household assets, it was challenging to assess the wealth quintile index for these patients. But relatively, they could estimate the monthly family income.
6. Given that chronic patients were heads of household; our findings were compared using the previous studies that addressed how satisfied household heads were with the CBHI schemes. On top of that, there was a lack of comparable data on satisfaction among chronic patients with CBHI.
7. **Enroller’s knowledge** of health insurance benefit package was corrected as **enrollee’s knowledge** of health insurance benefit package.
8. **N**o focus group discussions were used rather key informant interviewees from healthcare providers and CBHI beneficiaries who are community leaders were included.
9. The majority of important data were included in the manuscript but supplementary data such as questionaires and interview guides are included. The rest will be available upon reasonable request for readers.
10. **The figure** formats (.tiff format) with appropriate sizes have been attached.
