# Supplementary material for: Satisfaction of chronic patients with community-based health insurance schemes and related factors: Explanatory sequential mixed methods study
Source: PLOS Glob Public Health. 2024 Aug 7;4(8):e0003374. doi: 10.1371/journal.pgph.0003374 (PMC11305578; doi:10.1371/journal.pgph.0003374)
Supplement: Attachment — Submitted filename: Responses to Editors and Reviewers.docx [file pgph.0003374.s003.docx]

**Dear Editors and Reviewers in PLOS Global Public Health**

Thank you for your insightful feedback and for taking the time to read the manuscript number**, ‘PGPH-D-23-01081R1’** entitled as ‘**Satisfaction of Chronic Patients with Community-Based Health Insurance Scheme and Related Factors: Explanatory Sequential Mixed Methods Study**’**.**

The majority of the reviewers' and editors' comments were handled in the previous revision, and both reviewers agreed that the comments were addressed. **However, reviewer 1 merely voiced concerns about the reanalyzed data. Eleven variables remained after we thoroughly reanalyzed the data, and their adjusted odds ratios showed no discernible changes**. We respect Reviewer 1's concern, and we hope that this is the final edit.
